# Choledocholithiasis: Treatment Options in a Tertiary Care Setup in Pakistan

**DOI:** 10.7759/cureus.1587

**Published:** 2017-08-21

**Authors:** Ramlah Ghazanfor, Naeem Liaqat, Mehwish Changeez, Maham Tariq, Sara Malik, Khawaja R Ghazanfar, Jahangir S Khan

**Affiliations:** 1 Department of Surgery, Unit 1, Holy Family Hospital, Rawalpindi; 2 Department of Surgery, CMH Lahore Medical and Dental College

**Keywords:** choledocholithiasis, cholelithiasis, choledochodeudonostomy, choledochotomy, ercp

## Abstract

Introduction

Among patients with cholelithiasis, choledocholithiasis may also be present in about 18% of cases. They can be treated through various endoscopic, laparoscopic, and open surgical procedures.

Objective

The objective of this study was to determine the outcome of patients with choledocholithiasis being treated in our setup.

Methods

This descriptive case series was conducted at Holy Family Hospital, Rawalpindi, Pakistan over two years from January 2015 to December 2016. All patients with choledocholithiasis admitted to Surgical Unit 1 were included in this study. All patients underwent elective endoscopic retrograde cholangiopancreatography (ERCP). In patients with successful ERCP, laparoscopic or open cholecystectomy was performed at a later date. In patients in whom ERCP failed, open surgical clearance of the common bile duct (CBD), along with cholecystectomy, was done.

Results

A total of 200 cases of choledocholithiasis were admitted during the study period. Most of the participants (73%) in this study were female. Liver function tests were found to be deranged in 88 patients (44%) and normal in 112 patients (56%). At presentation, 3.5% (n=7) had concomitant acute biliary pancreatitis and 8% (n=16) had cholangitis. Successful ERCP followed by cholecystectomy was performed in 88.5% of cases. On the other hand, 11.5% (n=23) patients had failed ERCP due to impacted stones. They underwent open surgical procedures, i.e. 43.48% (n=10) had choledochotomies, 47.82% (n=11) had choledochoduodenostomies and 8.69% (n=2) had hepaticojejunostomies. No postoperative mortality was observed. However, anastomotic leaking occurred in 8.69% cases (n=2).

Conclusion

A two-staged procedure consisting of ERCP, followed by laparoscopic cholecystectomy, should be the first line of treatment for common bile duct (CBD) stones. In cases where ERCP fails, open surgical procedures still remain a relevant and a definitive option in resource-constrained setups.

## Introduction

Choledocholithiasis coexists in almost 18% of patients presenting with cholelithiasis. Over the last three decades, laparoscopic cholecystectomy and endoscopic retrograde cholangiopancreatography (ERCP) have established themselves as gold standard treatments for isolated gallbladder stones and common bile duct (CBD) stones, respectively. However, there remains a conflict of opinion in approach when they present simultaneously. Available options range from endoscopic, laparoscopic, and percutaneous approaches to open surgical techniques. Also, there is a lack of consensus whether these should be used alone, simultaneously, or in a sequential manner.

Generally, in well-equipped centres of the world, ERCP followed by laparoscopic cholecystectomy is recommended as a safe, effective, and economical procedure. However, various centres advocate that laparoscopic cholecystectomy with common bile duct (CBD) exploration or a Rendezvous technique where endoscopy and laparoscopy are performed simultaneously. They both have a similar efficacy profile with the added benefit of saving time [1]. With the advent of all above-mentioned technologies, open surgical procedures, such as CBD exploration or biliary-enteric bypasses, are now usually considered obsolete. However, various studies point them out to be a definitive option in cases where ERCP fails to retrieve stones or where facilities of advanced laparoscopic surgery are not available [2]. The objective of this study was to explore and assess treatment strategies employed in various cases of choledocholithiasis presenting in our setup and to determine their outcome.

## Materials and methods

After approval from the ethical committee, all patients of choledocholithiasis, admitted in Surgical Unit 1, Holy Family Hospital, Rawalpindi from the years 2015-2016 were included in this study (permitted by the Research and Ethical Committee, Rawalpindi Medical College and Allied Hospitals, RMC/PR_01/Jan 2015). The data were reviewed retrospectively. In each case, a definitive diagnosis was established by consultant general surgeons on the basis of history, clinical examination, liver function tests, and radiological investigations like ultrasound, computerized tomography (CT), and magnetic resonance cholangiopancreatography (MRCP). All patients were given treatment for obstructive jaundice, including intravenous antibiotics, hydration, vitamin K injections, and oral lactulose. Baseline investigations, including complete blood count, liver function tests, and coagulation profile, were obtained in all cases. All patients underwent elective ERCP by the consultant gastroenterologist. Those patients who had successful ERCP underwent laparoscopic or open cholecystectomy during the same admission. On the other hand, in patients whom ERCP failed, they underwent either choledochotomy or biliary-enteric drainage procedures like choledochoduodenostomy or choledochojejunostomy. Data were entered and analyzed using the Statistical Package for Social Sciences (SPSS), version 23.0 (IBM Corp., Armonk, NY). 

## Results

A total of 200 patients were managed for choledocholithiasis during the study period. Seventy percent of the patients were admitted via an outpatient department. Most of them were female (73%). The majority of patients (53%) were between 30-50 years old. Demographic features of all patients are given in Table 1.

**Table 1:** Demographic Details of Patients

ADMISSIONS	n (Percentage)
Outpatient	140 (70%)
Emergency	60 (30%)
Total	200 (100%)
GENDER	
Male	54 (27%)
Female	146 (73%)
AGE GROUPS	
<30 years	43 (21.5%)
30-50 years	104 (52%)
>50 years	53 (26.5%)

Only 29% of patients manifested clinical signs of obstructive jaundice like icterus, pruritus, or clay-colored stool. Presenting complaints are summarized in Table 2.

**Table 2:** Mode of Initial Presentation to Hospital

PRESENTATION	n (Percentage)
Incidental diagnosis on workup	43 (21.5%)
Clinical features of obstructive jaundice with normal liver function tests	58 (29%)
Isolated deranged liver function tests	76 (38%)
Cholangitis	16 (8%)
Acute biliary pancreatitis	7 (3.5%)

Liver function tests were found to be deranged in 88 patients (44%) and normal in 112 patients (56%). Among radiological investigations, ultrasound was the most commonly performed investigation, followed by MRCP and CT scan. These findings are listed in Table 3.

**Table 3:** Results of Various Radiological Investigations MRCP: magnetic resonance cholangiopancreatography; CT scan: computerized tomography scan

Investigation	Not done	Done	Stone detected
Ultrasound	5 (2.5%)	195 (97.5%)	135 (69.23%)
CT scan	178 (89%)	22 (11%)	11 (50%)
MRCP	154 (77%)	46 (23%)	24 (52.17%)
Endoscopic ultrasound	199 (99.5%)	1 (0.5%)	1 (100%)

All of these patients underwent ERCP on an elective basis. Mean procedure time was 37.84±27.89 minutes. Difficult cannulation was experienced in 18% of cases. The success rate of stone retrieval and CBD clearance was 88.5%. Post-ERCP pancreatitis was seen in 3% of cases. Cholangiogram findings are summarized in Table 4.

**Table 4:** Findings of Cholangiogram during ERCP (CBD: common bile duct, ERCP: endoscopic retrograde cholangiopancreatography)

Findings	n (Percentage)
Sludge retrieved	77 (38.5%)
Stone retrieved	100 (50%)
Large stone at confluence of right and left hepatic ducts	20 (20%)
Large stone impacted at distal CBD	80 (80%)
Large impacted stone that could not be retrieved	23 (11.5%)
Stent placed	11 (47.83%)
Stent not placed	12 (52.17%)

All patients in whom ERCP was successful (n=177) were offered same-admission cholecystectomy. Seventy-four percent had laparoscopic, and 26% had open cholecystectomy. The mean time interval between ERCP and cholecystectomy was 5±2.5 days. No major complications were seen in these patients during follow-up over the next six months.

Twenty-three (11.5%) patients had failed ERCP due to large, impacted stones. Fifteen (65.21%) of them were female. ERCP was reattempted in eight cases (34.7%). It failed to retrieve stones in all cases; a 10-French stent was placed every time to establish a free flow of bile. All 23 patients were offered open surgical procedures. On average, these patients had to wait 8±3.5 days for surgery. All procedures were performed by consultant surgeons. Average procedure time was 2.5±0.5 hours. The type of procedure performed is mentioned in Table 5.

**Table 5:** Various Types of Surgical Procedures Performed and Placement of T-tube

Procedure	n (Percentage)
Choledochotomy	10 (43.48%)
Choledochoduodenostomy	11 (47.83%)
Hepaticojejunostomy	2 (8.69%)
Total	23
T-tube placement	
Placed	8 (34.78%)
Not placed	15 (65.22%)

Postoperative mortality was nil. Fifty-five percent of patients were kept in high-dependency units until the second postoperative day. None of them needed intensive care unit (ICU) care. None of these patients suffered from sump syndrome. The average hospital stay after surgery was 7.5±10 days. Postoperative complications are summarized in Table 6. None of the patients reported recurrent jaundice, pain, incisional hernia, or had to undergo any further intervention at the one-year follow-up.

**Table 6:** Postoperative Complications

Events	n (Percentage)
Anastomotic leak	2 (8.69%)
Lower respiratory tract infection	3 (13.04%)
Wound infection	2 (8.69%)
Urinary tract infection	2 (8.69%)

## Discussion

ERCP has been accepted internationally as a first-line treatment option for choledocholithiasis with a success rate of almost 73%. In this study, the success rate was 88.5%. It failed in 23 patients, all of whom, invariably, had large, impacted stones. Eight of these patients underwent repeat ERCP, which proved to be futile every time. This contradicted the results of several international studies where stone-fragmentation and stenting, during an initial ERCP, helped to clear the CBD in further attempts [3].

Only 3% of patients suffered from post ERCP pancreatitis. This is much lower than the figure quoted in international studies [4]. All patients in whom ERCP was successful underwent same-admission cholecystectomy. Preoperative ERCP is believed to cause difficulty in the dissection of the triangle of Calot due to subsequent inflammation. This aspect, however, was not evaluated in this study.

In all cases of failed ERCP (n=23), open surgical clearance of the CBD was achieved via choledochotomy or biliary-enteric bypasses. The average delay in definitive surgery after failed ERCP was 8±3.5 days, mainly due to long operating lists/waits. Ten choledochotomies, 11 choledochoduodenostomies, and two hepaticojejunostomies were performed. With the development of endoscopic and laparoscopic techniques, treatment strategies for CBD stones have undergone a paradigm shift. ERCP alone or as a part of the Rendezvous technique has firmly established itself as a first-line treatment choice [5]. Surgical clearance of the CBD, once a common procedure, is now only considered only when ERCP fails. This is especially true in cases of open surgical procedures which now have largely been abandoned. However, there is mixed opinion about laparoscopic bile duct explorations, which several studies quote to be comparable to ERCP in terms of safety, morbidity, and cost-effectiveness. Several studies also show laparoscopic bile duct exploration or choledochoduodenostomy to be the procedure of choice in cases of failed ERCP. However, the need for expensive equipment and advanced surgical skills still preclude laparoscopic bile duct exploration from becoming popular in developing countries around the globe. Thus, many studies from Nepal, India, and Africa still state that open choledochotomies and biliary-enteric bypasses are practical options in dealing with large, impacted CBD stones.

There was no mortality in any case. The major morbidity included an anastomotic leak in two cases (8.69%), which were managed conservatively by keeping patients nil per oral (NPO) for a prolonged time, monitoring drain output, and CT scanning for any significant abdominal collection. No intervention was needed as patients improved. This complication rate, although not alarming, can be further reduced by opting for laparoscopic procedures that have fewer side effects [1, 6].

No evidence of sump syndrome, a potential complication after side-to-side choledochoduodenostomy, was detected in our study. Similar results were reported in a study published in 2011 [7]. Long-term complications could not be assessed in patients due to lack of proper follow-up.

Cost-effectiveness of ERCP and open surgical interventions could not be assessed satisfactorily for multiple reasons. First, no proper record of a patient or hospital expenditure on treatment was available for meaningful comparison. Moreover, all patients who underwent open surgery had first undergone a failed ERCP attempt for stone removal. This automatically lengthened their hospital stay and, therefore, increased expenditures as compared to those who had successful ERCP followed by a same-admission cholecystectomy. Internationally, cost-effective analysis establishes ERCP as far superior to surgical options [8]. However, at the same time, several studies stressed that open interventions can be useful in dealing with giant CBD calculi where ERCP may fail [9]. Currently, the only surgical procedure which can rival ERCP in terms of cost, as well as efficacy, is a single-staged laparoscopic procedure consisting of both cholecystectomy and CBD exploration [10-11]. This, unfortunately, could not be evaluated in our setup due to lack of advanced laparoscopic equipment and training.

This study had few limitations. The majority of enrolled patients were female (73%). This can be explained by the fact that gallstone diseases, including choledocholithiasis, are more common in females all over the world [12-13]. Also, this was a single-center study; therefore, results cannot be generalized.

## Conclusions

A two-staged procedure consisting of ERCP, followed by early laparoscopic cholecystectomy is an effective treatment for the majority of cases of choledocholithiasis. However, when ERCP fails, open choledochotomy or biliary-enteric bypass still remain relevant as definitive options in resource-constrained setups. Developing laparoscopic expertise in performing these procedures can minimize postoperative complications and shorten hospital stays.
